# Two cases of imported pneumonic plague in Beijing, China

**DOI:** 10.1097/MD.0000000000022932

**Published:** 2020-10-30

**Authors:** Haijiang Zhou, Shubin Guo

**Affiliations:** Emergency Medicine Clinical Research Center, Beijing Chao-yang Hospital, Capital Medical University & Beijing Key Laboratory of Cardiopulmonary Cerebral Resuscitation, Beijing, China.

**Keywords:** plague, streptomycin, *Yersinia pestis*

## Abstract

**Introduction:**

Plague is an acute, often fulminating infectious disease caused by *Yersinia Pestis* transmitted by rodents. It is rarely encountered in clinics, although natural plague foci are widely distributed around the world.

**Patient Concerns:**

A couple who are cattle and sheep herdsmen from the Inner Mongolia Autonomous Region presented with cough, expectoration and fever. The husband developed sudden onset of fever and bloody sputum after working the soil on his farm. The wife also developed fever after nursing his husband. Both patients were preliminarily diagnosed with severe pneumonia, but antimicrobial treatments in the local hospital were unsuccessful. Their conditions deteriorated and they were transferred to our center.

**Diagnosis:**

Preliminary etiological examinations were unremarkable, while blood and sputum specimens were found to be positive by RT-PCR and colloidal gold-immunochromatography assay targeting the F1 antigen and by reverse indirect hemagglutination assay. Pneumonic plague was confirmed.

**Interventions:**

Both patients were transferred to special infectious disease hospital for further treatment.

**Outcomes:**

The condition of the female patient deteriorated. The male recovered after treatment, while the female patient finally died.

**Conclusion:**

There are 3 main forms of plague: bubonic, pneumonic and septicemic. Humans can be infected by the bites of bacterium-bearing fleas or direct contact of wild animals that died from plague. Human plague can be transmitted by close contact through coughing droplet. Neglected diagnosis of plague could cause severe consequences. Strict surveillance and protection measures should be taken and the public should be alerted about potential risks when epizootic plague is detected.

## 1 Introduction

Plague, a zoonotic infection that has infected humans for thousands of years, is caused by *Yersinia pestis* (Y pestis) and is rarely encountered in clinics.^[1,2]^ The primary plague syndromes include bubonic, pneumonic and septicemic. Of them, pneumonic plague is the most severe manifestation of plague, with mortality rates approaching 100% without treatment.^[3]^ A neglected or delayed diagnosis will result in severe consequences. The rapid progression, lethality and ability to spread via aerosol of plague can raise panic among the public with the suspicion of intentional release of biological weapons.^[3]^ Nowadays, plague is still active in the natural foci worldwide. Recently, 2 cases of imported pneumonic plague were admitted in our hospital and were under proper treatment after diagnosis.^[4]^ Here, we report these 2 cases of imported pneumonia plague, which were the first 2 cases in Beijing in the recent 70 years, aiming at alerting clinicians to enhance awareness of any possible risks. The Institutional Review Board and Medical Ethics Committee have approved this study and Written Informed Consents were obtained from the patients.

## 2 Case presentation

A couple with the chief complaint of cough, expectoration and fever presented to the Emergency Department of Beijing Chao-yang Hospital, Capital Medical University on November 3, 2019. They are cattle and sheep herdsmen from the Sunite Zuo Qi (county) of the Xilinguole League (Prefecture) in Inner Mongolia Autonomous Region. Their past history was unremarkable.

The 43-year-old husband presented due to a 10-day history of cough, expectoration, bloody sputum, vomiting and fever. The highest temperature was 40 degree centigrade and oral antipyretics could reduce his fever. He developed sudden onset of fever, chills, vomiting, breathless and blood tinged-sputum on Oct. 25th, the day after working the soil on his farm. He visited the Sunite ZuoQi hospital on Oct. 25th and CT scan revealed lobar pneumonia, but 2 days of intravenous antimicrobial treatment did not reduce his symptoms. He still had fever, chills, cough, blood sputum, chest pain and vomiting. Re-examination of CT scanning revealed that his pneumonia aggravated. Suspected of influenza pneumonia, he was then transferred to Xilingol League Hospital by ambulance and was admitted in the intensive care unit (ICU), where he stayed for 7 days. However, his condition deteriorated after treatment. Suspected of severe influenza pneumonia, he was transferred to Emergency Department of Beijing Chao-yang Hospital, Capital Medical University by ambulance. Preliminary test result of nasopharyngeal swab for influenza screen was negative in the fever clinic and then he visited the emergency department.

**Figure 1:**
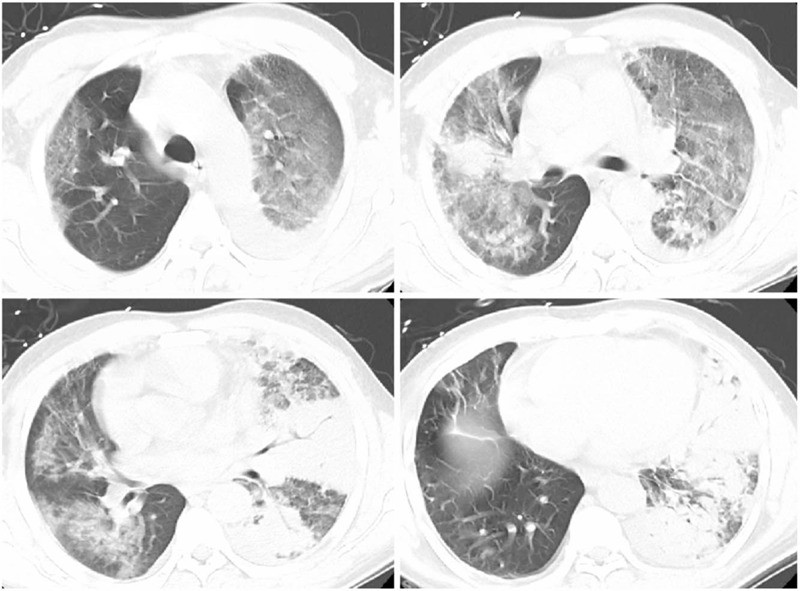
CT Scan of the male patient on admission revealed multiple patchy consolidation and ground glass opacity in bilateral lungs. CT = computed tomography.

Physical examination on admission revealed a body temperature of 36.3°C, a pulse rate of 74 times/min, a respiratory rate of 20 times/min and a blood pressure of 126/85 mm Hg. He was conscious with poor spirits. Auscultation revealed rough breathing sound with wet rales and regular heart rhythm. No swelling lymph node was found in axillary and inguinal regions. There was a flat and soft abdomen without tenderness. Blood routine test revealed a white blood cell count of 28.6 × 10^9^/L, a neutrocyte count of 26.53 × 10^9^/L, a lymphocyte level of 1.26 × 10^9^/L, a monocyte level of 0.68 × 10^9^/L, a hemoglobin level of 132 g/L and a platelet count of 267 × 10^9^/L. Blood biochemistry revealed an albumin level of 29.1 g/L, an alanine aminotransferase level of 80U/L, an aspartate aminotransferase level of 29 U/L, a total bilirubin level of 22.6 umol/L, a direct bilirubin level of 11.0 umol/L, a creatinine level of 44.5 umol/L, a blood urea nitrogen of 3.18 mmol/L, a troponin level of 0.01 ng/ml, a creatine kinase isoenzyme level of 0.7 ng/ml, a potassium level of 3.7 mmol/L, a sodium level of 135.7 mmol/L and a blood sugar level of 8.6 mmol/L. Arterial blood gas analysis revealed PH 7.49, lactate 1.1 mmol/L, PaO_2_ 69.1 mmol/L, PaCO_2_ 27.2 mm Hg, HCO_3_^-^ 21 mmol/L, base excess of -0.5 mmol/L and PaO_2_/FiO_2_ 330.4 mmol/L on oxygen storage mask (oxygen saturation 100%, 10L/min). Coagulation test revealed prothrombin time of 12.8 s, prothrombin activity 80.3%, international normalized ratio of 1.12, fibrinogen of 464.5 mg/dL and D-dimer of 4.98 mg/L FEU. The erythrocyte sedimentation rate was 17 mm/h (normal range 2–15 mm/h). The procalcitonin level was 0.18 ng/mL and C-reactive protein was 11.30 ng/dL (normal range 0–0.8 ng/dL). The brain natriuretic peptide was 165 pg/mL (normal range 0–100 pg/mL). CD3 T cell was 71.7%, CD4+ T cell was 41.5% and CD8+ T cell was 24.6%. Electrocardiogram showed sinus rhythm with a heart rate of 75 times/min. High recognition CT scan of the lung revealed multiple patchy consolidation and ground glass opacity in bilateral lungs (Fig. 1).

The 46-year-old wife accompanied and kindly nursed his husband in the course of his illness and handled his vomitus in the spittoon. She developed cough, expectoration, bloody sputum, fever and chills on October 31. She also had fatigue, chest pain, nausea and vomiting. The highest temperature was 38°C. Chest CT scan in the Xilingol League Hospital revealed pneumonia and she was also hospitalized. Intravenous antimicrobial treatment did not show significant improvement. She was also transferred to Beijing Chao-yang Hospital, Capital Medical University. Preliminary test result of nasopharyngeal swab for influenza screen was also negative in the fever clinic.

**Figure 2:**
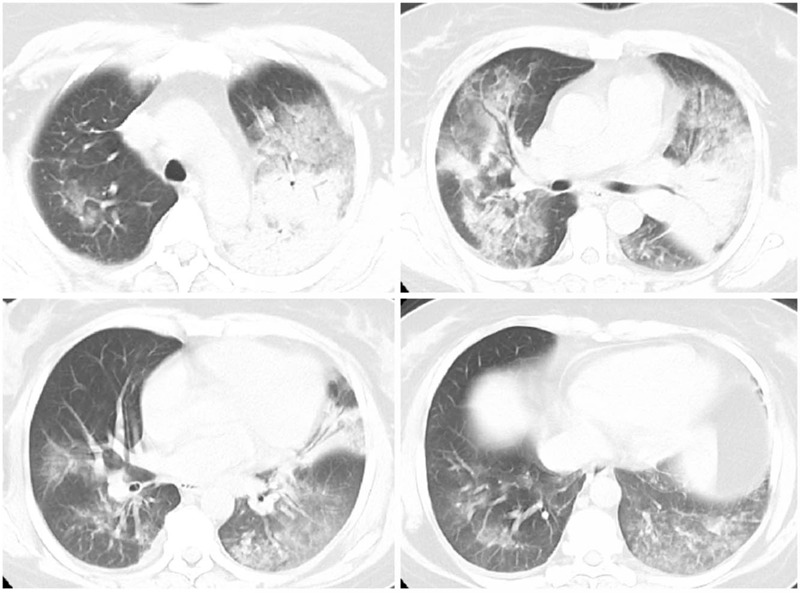
CT Scan of the female patient on admission revealed multiple patchy consolidation shadows and air bronchgram in bilateral lungs. CT = computed tomography.

Physical examination on ED arrival revealed a body temperature of 37.3°C, a pulse rate of 101 times/min, a respiratory rate of 22 times/min and a blood pressure of 108/69 mm Hg. She was conscious with poor spirits. Auscultation revealed rough breathing sound with wet rales and regular heart rhythm. There was a flat and soft abdomen without tenderness. Blood routine test revealed a white blood cell count of 9.76 × 10^9^/L, a neutrocyte count of 8.70 × 10^9^/L, a lymphocyte level of 0.84 × 10^9^/L, a monocyte level of 0.18 × 10^9^/L, a hemoglobin level of 117 g/L and a platelet count of 134 × 10^9^/L. Blood biochemistry revealed an albumin level of 36.6 g/L, an alanine aminotransferase level of 22U/L, an aspartate aminotransferase level of 29 U/L, a total bilirubin level of 12.1 umol/L, a direct bilirubin level of 4.5 umol/L, a creatinine level 50.6 umol/L, a blood urea nitrogen 3.72 mmol/L, a troponin level of 0.00 ng/ml, a creatine kinase isoenzyme level of 0.2 ng/mL, a potassium level of 2.9 mmol/L, a sodium level of 136 mmol/L and a blood sugar level of 6.3 mmol/L. Arterial blood gas analysis revealed PH 7.44, lactate 0.80 mmol/L, PaO_2_ 59.5 mmol/L, PaCO_2_ 26 mm Hg, HCO_3_^-^ 17.8 mmol/L, base excess of -4.3 mmol/L and PaO_2_/FiO_2_ 284.8 mmol/L on oxygen storage mask (oxygen saturation 100%, 10L/min). Coagulation test revealed prothrombin time of 11.9 s, prothrombin activity 89.1%, international normalized ratio of 1.03, fibrinogen of 738 mg/dL and D-dimer of 3.12 mg/L FEU. The procalcitonin level was 2.67 ng/mL and C-reactive protein was 11.30 ng/dL (normal range 0–0.8 ng/dL). The brain natriuretic peptide was 76 pg/mL (normal range 0–100 pg/mL). Electrocardiogram showed sinus rhythm with a heart rate of 102 times/min. high recognition CT scan of the lung revealed multiple patchy consolidation shadows and air bronchgram in bilateral lungs (Fig. 2).

**Figure 3:**
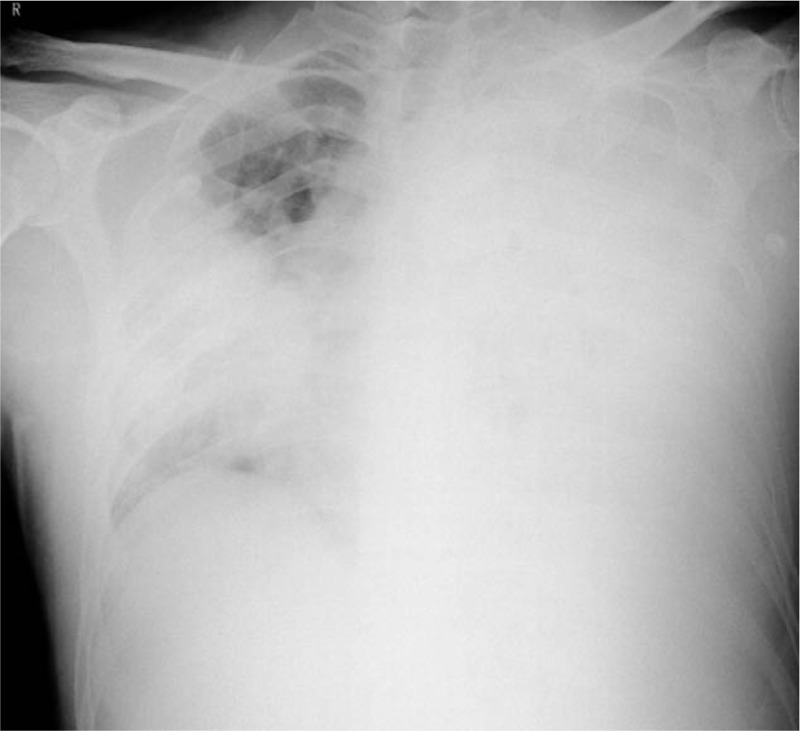
Chest X-ray of the male patient after 1 week of treatment revealed consolidations on bilateral lungs. CT = computed tomography.

**Figure 4:**
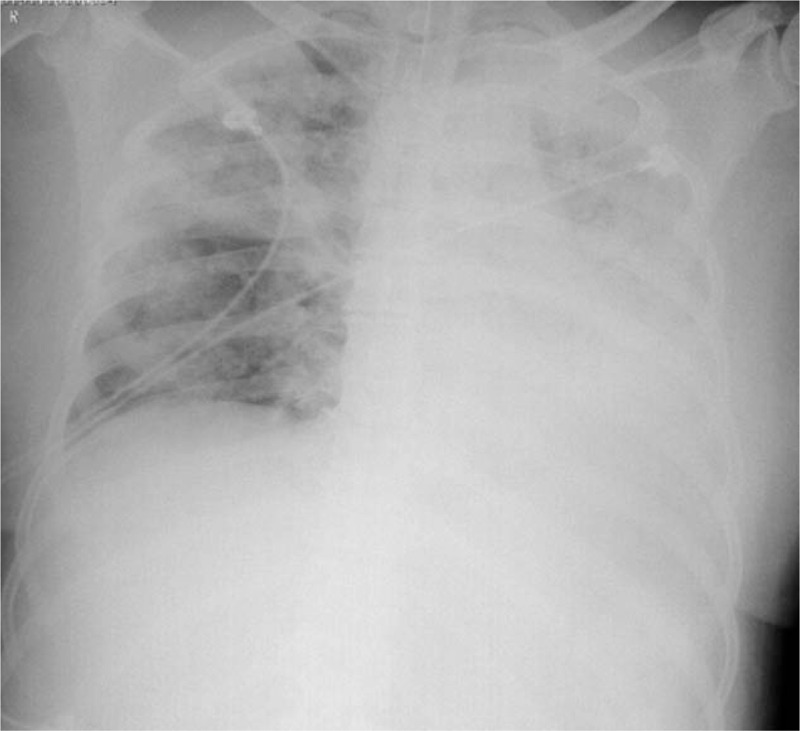
Chest X-ray of the female patient after 1 week of treatment revealed consolidations on the left lung and patchy shadow on the right lung. CT = computed tomography.

Both patients were presumably diagnosed with severe community-acquired pneumonia and received oral Oseltamivir and intravenous moxifloxacin and cefoperazone and sulbactam for injection. They were quarantined and admitted in the negative pressure quarantine wards of the respiratory intensive care unit. Suspected of viral pneumonia, medical staff in respiratory intensive care unit took strict protective measures. Both patients received intermittent use of noninvasive mechanical ventilation and high-flow nasal canula oxygen therapy. Bronchoscope examinations were taken and sputum and bronchoalveolar lavage fluid samples were sent for etiological examinations. The results were unremarkable except positive results for mycoplasma IgG antibody, Epstein-Barr virus antibody and Klebsiella pneumoniae in the male. The condition of the female patient deteriorated and invasive mechanical ventilation through endotracheal intubation was provided on Nov. 8th. However, on November 12, blood and sputum specimens were found to be positive by RT-PCR and colloidal gold-immunochromatography assay targeting the F1 antigen and by reverse indirect hemagglutination assay.^[4]^ Although *Y pestis* was not able to be isolated by culture, NGS sequencing was weakly positive for *Y pestis* genetic material.^[4]^ Pneumonic plague was confirmed and the 2 patients were treated in isolation. The condition of the male patient improved after treatment (Fig. 3). However, the condition of the female patient deteriorated and she received V-V extracorporeal membrane oxygenation treatment on November 12 before transferred to special hospital for infectious disease (Fig. 4). The male patient recovered after treatment, while the female patient finally died. A total of 447 persons with direct contact in Beijing and 46 in Inner Mongolia were taken strict isolation measures, quarantined for medical observation and taken preventive medication therapy.^[4]^ All persons with close contact were discharged from medical observation on November 21, 2019.

## 3 Discussion

Plague is an acute vector-borne infectious disease caused by *Yersia. Pestis (Y pestis)* and transmitted by fleas to a variety of wildlife rodents representing natural reservoirs for the disease.^[5]^ Plague has caused 3 major pandemics in human history and has influenced the path of human civilization.^[3]^*Y pestis*, a Gram-negative bacterium belongs to the family *Enterobacteriaceae*, was discovered and isolated by the Institut Pasteur bacteriologist Alexandre Yersin during the third pandemic plague in Hong Kong in 1894.^[5,6]^*Y pestis*, which is currently recognized as a clonally expanded genomically degenerating variant of *Yersinia pseudotuberculosis*, evolved from *Yersinia pseudotuberculosis* about 5000 to 7000 years ago by obtaining a flea-transmitted life cycle and the ability to cause host systemic infection.^[2]^ The pCD1, a *Yersinia pseudotuberculosis*-inherited plasmid, and 2 kinds of newly acquired plasmids (pMT1 and pPCP1), play a crucial role in the pathogenicity of *Y pestis*.^[2]^ It is now recognized that a type III secretion system (T3SS), which is a needle-like structure on the bacterial surface and encoded by inherited plasmid pCD1, is essential for the pathogenesis of *Y pestis* through all routes of infection.^[2,3]^ If functional T3SS is absent, the growth of *Y pestis* will decline in the lung.^[3]^ T3SS can inject toxic Yersinia outer protein into host cells when pathogens come into contact with cells.^[2,7]^ Yersinia outer protein can block the host's innate immunity, damage host cell structures, down-regulate the production of pro-inflammatory cytokines and induce cell death by multiple mechanisms, playing significant roles in plague pathogenesis.^[2,5]^ Delivery of Yop effectors and cell invasion require adhesion of *Y pestis* to host cells.^[5]^ The well-known fraction 1 (F1) capsular antigen, encoded by plasmid pMT1, prevents bacterial uptake by inhibiting adhesion and is now utilized as a vaccine component and a diagnostic target.^[2,5]^ In addition, the pPCP1, another newly acquired plasmid, encodes plasminogen activator (Pla) which is very crucial for bacterial invasion into tissues.^[2,8]^

There are 3 major forms of plague, including bubonic, pneumonic and septicemic plague.^[2,9]^*Y pestis* can be transmitted by flea bites (causing bubonic plague), respiratory droplet (causing pneumonic plague), consumption of uncooked contaminated meat (causing gastrointestinal plague) as well as contact with infected animals (causing conjunctivitis, skin plague or pneumonic plague).^[2]^ Delayed recognition and diagnosis of bubonic plague can progress into pneumonic plague or septicemic plague, while septicemic plague can also be caused by blood infection of *Y pestis* through a wound cut.^[2]^ Primary pneumonic plague is caused by aerosol exposure to, while secondary pneumonic plague develops from dissemination of *Y pestis* into the lungs during bubonic or septicemic plague.^[3]^

Plague is a biphasic disease determined by host immune response and disease pathology.^[3]^ One of the most distinguishing characteristics of *Y pestis* infection, either through a flea bite or through respiratory droplet exposure, is the brutal conversion from an absence of immune response and clinical symptoms to a bursting inflammation and fatal sepsis with abundant bacteria in the body.^[5]^ The disease progression pattern resembles what is seen in humans and has been demonstrated in animal models.^[3]^ This lag period was termed as pre-inflammatory phase and help *Y pestis* reproduce silently.^[5]^ An abrupt transition happens about 36–48 hours after infection, when the “pro-inflammatory phase” of the disease starts.^[3]^ This is a critical turning point that invariably results in death, unless treated promptly.^[3]^

Pneumonic plague differed from bubonic plague by the mode of infection, the target tissue and pathophysiology and lethality.^[5]^ The initial symptoms of plague resembles those of influenza with high fever, chills, malaise and headache.^[2]^ An important clue for suspected case of plague is the contact history with wild animals in natural plague foci or other patients with plague.^[2]^ If a patient presented sudden onset of fever after contact with dead rodents from plague foci, plague should be highly suspected.^[2]^ Patients with bubonic plague can presented with regional lymph node swelling, pneumonic plague can present with severe coughing and pneumonic signs by X-ray, while septicemic plague can present with sudden high fever and chills.^[2]^ The incubation period is 2 to 3 days or as long as 6 days.^[10]^ Patients inhaling a large quantity of *Y pestis* could have an incubation period of 1 day or less and their signs and symptoms could progress very quickly.^[10]^ Patients with bubonic plague could present with regional hot skin with progressive pain in the flea-biting region and forced position caused by swollen lymph nodes.^[2]^

Plague should be classified as a suspected, presumptive or confirmed case according to the guideline released by WHO.^[2,11]^ For suspected patients, compatible symptoms and signs should be accompanied by epidemiological characteristics including a trip to an area endemic for plague within 10 days before the onset of symptoms and signs, contact history to plague patients or animals and/or obvious history of flea bites.^[2]^ For a presumptive case, except for characteristics depicted above, the patient should exhibit the following criteria: Giemsa or Wayson staining of Gram-negative coccobacilli in samples from bubo aspirate, blood or sputum, with bipolar appearance suggestive of a safety pin; detection of F1 antigen from bubo aspirate, blood or sputum; detection of serum F1 antibody without history of previous plague infection or immunization; and PCR detection of *Y pestis* in bubo aspirate, blood or sputum.^[2]^ For a confirmed case, except for meeting criteria of a suspected case, the following criteria should be included: *Y pestis* isolated from bubo aspirate, blood or sputum; *Y pestis* identified by morphological, biochemical, phage lysis, F1 antigen detection and PCR test; and 4-fold increase in anti-F1 antibody titer in paired serum samples.^[2]^ The gold standard for diagnosis of plague in laboratory is the isolation and identification of plague pathogen from clinical specimen.^[2]^ Nowadays, F1 antigen is typically utilized as a target to detect *Y pestis* by immunological methods.^[2]^

Early identification and timely administration of effective antibiotics are the keys to successful treatment of plague.^[2]^ It would be fatal if administration of effective antibiotics and anti-shock therapy are delayed for more than 24 hours.^[2]^ Most of the isolated *Y pestis* worldwide are sensitive for streptomycin, while a multidrug resistant strain was previously reported in Madagascar.^[12]^ Streptomycin and gentamicin are recommended for adult patients, including immunocompromised patient and pregnant women.^[2]^ Dosage of streptomycin and gentamicin should be reduced in children. Moreover, the combination of doxycycline, ciprofloxacin and chloramphenicol could also be administered in both adult and children.^[2]^ However, regulations of administration of antibiotics vary in different countries.

Patients with bubonic plague without secondary pneumonic plague and septicemic plague have a very low risk of transmission, while patients with primary or secondary pneumonic plague can transmit *Y pestis* to close contacts by coughing droplet.^[2]^ The first 24 hour since the onset of plague is non-infectious,^[13]^ however, patients with bloody expectoration are highly infectious. Wearing a face mask or evening covering one's mouth by a jacket can effectively prevent transmission of plague.^[2,9]^ It is important to emphasize that patients with suspected pneumonic plague or bubonic plague with secondary pneumonic plague or septicemic plague should be strictly isolated.^[2,9]^

There are 4 plague foci in Inner Mongolia of China and the last human plague case was reported in 2007 due to skinning a dead hare.^[4]^ The couple reported in this article lived in grassland and the male patient most likely got infected while working the soil on his farm, where *M. unguiculatus* serves as a primary plague host.^[4]^ A large rodent die-off was observed in their living area, raising the possibility that the decayed bodies in the dirt or in rat holes could have produced infectious aerosols.^[4]^

Plague is rarely encountered in clinics, negligence and delayed diagnosis can lead to severe consequences. Clinicians in emergency department should enhance their awareness and must be alert to pay special attention to this deadly disease. Public education efforts should focus on enhancing surveillance and improving personal protection measures. When epizootic plague is detected, local medical staff and the public should be alerted the potential risks of plague.

## Author contributions

**Conceptualization:** Haijiang Zhou, Shubin Guo.

**Data curation:** Haijiang Zhou.

**Formal analysis:** Haijiang Zhou.

**Investigation:** Haijiang Zhou, Shubin Guo.

**Methodology:** Haijiang Zhou, Shubin Guo.

**Supervision:** Shubin Guo.

**Validation:** Shubin Guo.

**Writing – original draft:** Haijiang Zhou.

**Writing – review & editing:** Haijiang Zhou, Shubin Guo.
